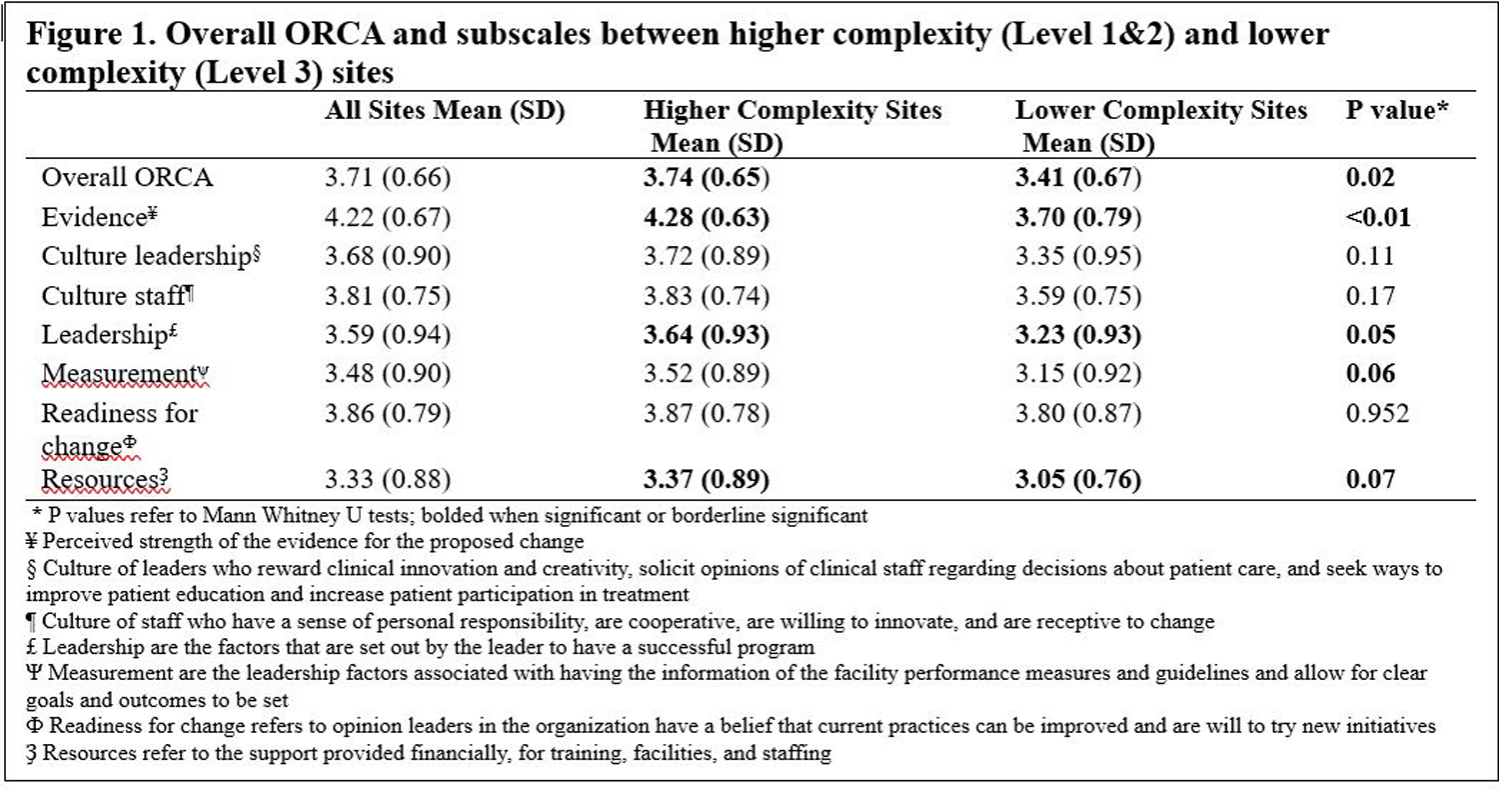# Organizational Readiness for Change Depends on Facility Complexity When Developing a National Stewardship Intervention

**DOI:** 10.1017/ash.2024.163

**Published:** 2024-09-16

**Authors:** Eva Amenta, Larissa Grigoryan, Sophia Braund, David Ramsey, John Donnelly, Rogelio Hernandez, Aanand Naik, Barbara Trautner

**Affiliations:** Baylor College of Medicine

## Abstract

**Introduction:** The organizational readiness for change assessment survey (ORCA) is a tool to assess a site’s readiness for implementation and identify barriers to change. As the “Kicking CAUTI” antibiotic stewardship intervention rolled out on a national scale, we administered ORCA surveys to participating sites to capture baseline actionable information about differences among sites, to inform implementation. **Methods:** ORCA surveys were distributed by email to prescribing providers, nurses, pharmacists, infection preventionists, and quality managers at 40 participating VA Hospitals. VA hospital sites who submitted three or more surveys and their complexity level (measured as Level 1 (highest)-3) were included in the analysis. The highest complexity level facilities are those with the largest patient volume/risk, teaching and research, along with the largest number of physician specialists and contain at least five ICUs. Mean Likert scores were calculated for each of the 7 ORCA subscales on a scale of 1-5 (5 highest), and the mean of the 7 subscales was the overall ORCA score for a site. Non-parametric testing was performed comparing overall ORCA and each subscale based on complexity. **Results:** Among the participating sites, 30/40 (75%) completed at least three surveys, with a total of 202 surveys included for analysis, with 82% of surveys coming from higher complexity centers (Level 1). The highest ranked ORCA domain was the evidence subscale (measures perceived strength of evidence), mean 4.2, (SD 0.7). The lowest ranked ORCA domain across sites was resources (available to facilitate implementation), mean 3.3 (SD 0.9). Higher complexity centers had a significantly higher overall ORCA score than lower complexity centers (Level 1 or 2 vs. Level 3, p= 0.02). This difference was driven by the subscales evidence (p < 0 .01), leadership (p =0.05), measurement (p= 0.06), and resources (p=0.07) all being higher in the higher complexity facilities (Figure 1). Two of the categories (leadership and measurement) pertain to an organization’s leaders ability to create an environment for change to occur as well as promoting team building. **Conclusions:** The lowest scoring ORCA domain across all sites was the respondents’ perception of resources (staff, training) available for achieving change. Perceived resources were also lower in lower complexity sites, implying that medical centers of lower complexity may have higher barriers when implementing an antimicrobial stewardship intervention. This finding highlights the benefit of a national stewardship campaign that provides support to lower complexity medical centers that may not otherwise receive targeted training and support for their efforts.

**Disclosure:** Barbara Trautner: Stock: Abbvie--sold in December 2023; Abbott Laboratories--sold in December 2023; -Bristol Myers Squibb--sold in December 2023; Pfizer--sold in December 2023; Consultant--Phiogen--consultant. Contracted research through NIAID for STRIVE trial, currently testing Shionogi product; Contracted research--Peptilogics; Contracted research—Genentech

**Figure:**